# The socio-matrix reloaded: from hierarchy to dominance profile in wild lemurs

**DOI:** 10.7717/peerj.729

**Published:** 2015-01-15

**Authors:** Ivan Norscia, Elisabetta Palagi

**Affiliations:** 1 Natural History Museum, University of Pisa, Calci, Pisa, Italy; 2 Institute of Cognitive Sciences and Technologies, Unit of Cognitive Primatology & Primate Center, CNR, Rome, Italy

**Keywords:** Lemurs, Sifaka, Steepness, Linearity, Strepsirhines, Prosimians, Madagascar, Social management, Transitivity

## Abstract

Dominance hierarchy influences the life quality of social animals, and its definition should in principle be based on the outcome of agonistic interactions. However, defining and comparing the dominance profile of social groups is difficult due to the different dominance measures used and because no one measure explains it all. We applied different analytical methods to winner-loser sociomatrices to determine the dominance profile of five groups of wild lemurs (species: *Lemur catta*, *Propithecus verreauxi*, and *Eulemur rufus* x *collaris*) from the Berenty forest (Madagascar). They are an excellent study model because they share the same habitat and an apparently similar dominance profile: linear hierarchy and female dominance. Data were collected over more than 1200 h of observation. Our approach included four steps: (1) by applying the binary dyadic dominance relationship method (I&SI) on either aggressions or supplant sociomatrices we verified whether hierarchy was aggression or submission based; (2) by calculating normalized David’s scores and measuring steepness from aggression sociomatrices we evaluated whether hierarchy was shallow or steep; (3) by comparing the ranking orders obtained with methods 1 and 2 we assessed whether hierarchy was consistent or not; and (4) by assessing triangle transitivity and comparing it with the linearity index and the level of group cohesion we determined if hierarchy was more or less cohesive. Our results show that *L. catta* groups have got a steep, consistent, highly transitive and cohesive hierarchy. *P. verreauxi* groups are characterized by a moderately steep and consistent hierarchy, with variable levels of triangle transitivity and cohesion. *E. rufus* x *collaris* group possesses a shallow and inconsistent hierarchy, with lower (but not lowest) levels of transitivity and cohesion. A multiple analytical approach on winner-loser sociomatrices other than leading to an in-depth description of the dominance profile, allows intergroup and cross-species comparisons.

## Introduction

In social animals, an individual’s status in its dominance hierarchy can dramatically influence its life quality, including general health, stress levels, resource access, and reproductive potential (Preuschoft & van Schaik, 2000; Sapolsky, 2005). From a social perspective, dominance rank scaffolds the quality of inter-individual relationships and permeates all behavioral spheres (including aggression, affiliation, parental care, and sexual activity) (Clutton-Brock, Albon & Guinness, 1984; Ogola Onyango et al., 2008; Palagi, Chiarugi & Cordoni, 2008; Norscia, Antonacci & Palagi, 2009). From an ecological perspective, the structure of dominance relationships can influence reproductive success (Pusey, Williams & Goodall, 1997; von Holst et al., 2002), resource access (Clutton-Brock, 1982; Krebs & Davies, 1987), territory quality (Fox, Rose & Myers, 1981), predation risk (Hall & Fedigan, 1997), and energy budgets (Isbell & Young, 1993; Koenig, 2000).

Drews (1993) pointed out that the definitions of dominance could be based on theoretical constructs or on certain observable behaviors, and focus on different parameters, such as dyads or single individuals, physical properties of subjects or their role, aggressive encounters or the lack of them. Indeed, the definition of dominance has been based on the confrontation of individuals in agonistic interactions (e.g., Bernstein, 1981; Leiva & de Vries, 2011) and on other correlates, depending on species-specific behavioral repertoire (e.g., direction of approach-retreats, priority of access, special position, and genital display; de Waal & Luttrell, 1985; Cheney, 1977; Kitchen, Cheney & Seyfarth, 2005; Parr et al., 1997; Murray, 2007; Frank, 1986; Hirsch, 2010; Lemel & Wallin, 1993; Alvarez, 1975).

Within social groups, hierarchies can be either linear (A > B > C > D) or non linear (e.g., triangular: A > B and B > C but C > A, pyramidal: A>[B = C = D], or class system based: [A + B] > [C = D + E + F]). Such feature derives from relational properties of networks of dyads rather than from properties of individuals or single dyads (Preuschoft & van Schaik, 2000). In particular, linear hierarchy derives from a set of binary dominance relationships and depends on the number of established relationships and on the degree to which they are transitive (Landau, 1951; Kendall, 1962; Appleby, 1983; de Vries, 1995). The degree of linearity can be measured via the corrected Landau’s index (*h*′; Landau, 1951), which has been used to determine the structure of dominance relationships in social groups and make comparisons (Palagi, Antonacci & Norscia, 2008; Paoli & Palagi, 2008; Hewitt, Macdonald & Dugdale, 2009). However, hierarchies sharing similar levels of linearity (*h*′) can differ in the extent of power asymmetry between individuals (Flack & de Waal, 2004). For this reason de Vries, Stevens & Vervaecke (2006) introduced the concept of steepness, another property of dominance hierarchy. The steepness derives from the size of the absolute differences between adjacently ranked individuals in their overall success in winning dominance encounters. When these differences are large the hierarchy is steep; when they are small the hierarchy is shallow. While linearity (based on the binary dyadic dominance relationships) derives from the direction of power asymmetry, steepness requires a cardinal rank measure and considers the extent of power asymmetry (Flack & de Waal, 2004; de Vries, Stevens & Vervaecke, 2006). However, as pointed out by de Vries, Stevens & Vervaecke (2006), the comparison of the hierarchical structure of different groups using the steepness values has a limitation related to the presence of dyads for which zero interactions were recorded. As it has been shown by Klass & Cords (2011) using both simulated and empirical data from four wild monkey groups, the steepness measure is negatively influenced by the proportion of zero dyads in the matrix. If the zero dyads accurately reflect the absence of clear dominance-subordination relationships among individuals, interpreting lower steepness as an indication of less despotic hierarchy is correct. On the contrary, when these zero dyads are due to observational problems, this interpretation is questionable (de Vries, Stevens & Vervaecke, 2006).

To avoid the problems related to zero dyads, Shizuka & McDonald (2012) and Shizuka & McDonald (2014) presented a new measure—based on network structural properties—for determining the level of hierarchy transitivity, less sensitive to observational zeros. This measure, called triangle transitivity (*t*_tri_) is based on the transitivity of dominance relations among sets of three individuals that all interact with each other. Triangle transitivity and linearity are essentially equivalent when the dominance relations of all dyads are known but—as discussed above—such conditions are not always met (Shizuka & McDonald, 2012). The method by Shizuka & McDonald (2012) follows a logic similar to that of de Vries (1995), but the procedure is conducted without filling in zero dyads with randomized dominance relations. In fact, filling in zero dyads artificially decreases the level of linearity because it creates cyclic (and not transitive) triads, e.g., A dominates B, B dominates C, and C dominates A (A >B >C >A). According to the framework presented above, it is clear that different aspects of dominance hierarchy can be distinguished that rely on different parameters, thus providing different outcomes. For the first time, we systematically combine different measures into a stepwise approach in order to verify how and whether they add to a more comprehensive definition of the dominance profile of social groups.

As a model for our investigation, we used five wild groups of three sympatric strepsirhine species (*Lemur catta*, *Propithecus verreauxi*, and *Eulemur rufus* x *collaris*) which share the same habitat and part of their home range in the Berenty forest (south Madagascar) and show similar social system features. In fact, they are characterized by multimale-multifemale group composition, linear hierarchy, and exclusive female dominance over males (Norscia & Palagi, 2011; Palagi & Norscia, 2011; Sclafani et al., 2012; Palagi, Antonacci & Norscia, 2008). Below, we describe the four steps of the methodological procedure applied in this study. For each step, we formulate predictions on lemurs aimed at assessing whether our approach is able to unveil differences in the dominance profile of social groups whose social system seems alike.

Step 1: In primates, either avoidance or aggression have been used to determine the dominance hierarchy (Watts, 1994; Pruetz & Isbell, 2000; Radespiel & Zimmermann, 2001; Cooper & Bernstein, 2008). By running the same test on both avoidance and aggression sociomatrices, based on I&SI rank orders (de Vries, 1998), this step allows one to detect if hierarchy linearity is established also via submission patterns other than via overt aggressions.

*Lemur catta* groups are matrilines with strict dominance hierarchies and are characterized by the presence of formalized subordination vocalizations (Jolly, 1966; Kappeler, 1999; Pereira, 2006; Koyama et al., 2001). *Propithecus verreauxi* possesses subordination signals (e.g., submissive chatters) but also a linear hierarchy based on aggression (Kappeler, 1999; Lewis & van Schaik, 2007; Palagi, Antonacci & Norscia, 2008). In this species, aggression by subordinate males toward the dominant males often occur simultaneously with submissive signals (Lewis & van Schaik, 2007). *Eulemur fulvus* seems not to possess formalized subordination signals (Kappeler, 1999; *Eulemur fulvus* subspecies have been later accorded species status, including *E. rufus* and *E. collaris*; Mittermeier et al., 2008). *E. rufus* x *collaris* in Berenty can have a linear hierarchy based on aggressions (Palagi & Norscia, 2011). In the light of this framework, we expect that *L. catta* and *P. verreauxi* groups, but not the group of *E. rufus* x *collaris*, may establish a linear hierarchy also using submissive behaviors (avoidance, in this study) (Prediction 1).

Step 2: By using a cardinal rank measure (based on normalized David’s scores, see methods) and considering the extent of power asymmetry between individuals (Flack & de Waal, 2004; de Vries, Stevens & Vervaecke, 2006), this step allows the evaluation of hierarchy steepness of social groups.

Dominance steepness was qualitatively defined as despotic for *L. catta*, egalitarian for *P. verreauxi* and unclear for *E. fulvus* spp. (Kappeler, 1999). Therefore, the groups of *P. verreauxi* and *E. rufus* x *collaris* might show less steep hierarchies compared to *L. catta* groups (Prediction 2).

Step 3: Although different, linearity and steepness both rely on the outcome of aggressive encounters between group members (de Vries, Netto & Hanegraaf, 1993; de Vries, Stevens & Vervaecke, 2006). By comparing the hierarchy obtained via binary dyadic relationships and via normalized David’s scores (the two analytical tools used for determining linearity and steepness), this step allows one to detect if the hierarchy remains consistent between methods.

We expect to find higher consistency in *Lemur catta* than in other groups because—based on the information provided at steps 1 and 2—*L. catta* groups normally have a strict hierarchy established via submissive signals and aggression (Prediction 3).

Step 4: By comparing triangle transitivity (Shizuka & McDonald, 2012) with the linearity measures, we evaluate the impact that non interacting dyads have on different aspects of dominance hierarchy. By associating this information with the measure of group cohesion around the dominant, we assess whether dominance hierarchy was more or less “cohesive”; that is, more or less influenced by individuals’ spatial dispersal.

*L. catta* and P. *verreauxi* form compact groups, defined as “troops” (Jolly, 1966) and “foraging units” (Richard, 1985), respectively. Instead, in *Eulemur* spp both males and females show low cohesion levels (Kappeler, 1999). Thus, we expect individuals’ dispersal to affect hierarchy transitivity more in *Eulemur rufus* x *collaris* than in the groups of the other study species (Prediction 4).

## Methods

### Ethics statement

Because the study was purely observational the Animal Care and Use board (University of Pisa) waives the need for a permit. The study was conducted with no manipulation of animals. The study was carried out in the private Berenty Reserve (South Madagascar). The owners Mr De Heaulme (and family) permitted us to conduct the observational study.

### Study site, groups, and data collection

This study was performed in the Berenty forest (South Madagascar, S 25.00°; E 46.30°). The site is characterized by two main climatic periods: a wet season from October to March and a dry season from April to September (Jolly et al., 2006). We observed animals of three sympatric species, and in particular two groups (A and B) of *Lemur catta* (ring-tailed lemurs), two groups (A and B) of *Propithecus verreauxi* (Verreaux’s sifaka), and a single group of introduced *Eulemur rufus* x *collaris* (brown lemurs). Group composition is reported in Table 1. In the study we considered both adults and subadults, determined on the basis of scent marking frequency and body size (Kappeler, 1998; Palagi, Gregorace & Borgognini Tarli, 2002; Jolly, 1966).

**Table 1:** Data collection information. Table listing group composition, observation period, and time of focal observations.

Study groups	Group composition (age, sex)	Observation period	Observation time
*Lemur catta* A	6 AF, 3 AM, 1 SAM	March–July 2008	160 hs total; approx. 16 hs/ind
*Lemur catta* B	6 AF, 5 AM, 2 SAF	March–July 2008	229 hs total; approx. 18 hs/ind
*Propithecus verreauxi* A	2 AF, 7 AM, 1 SAF	November 2006–February 2007	400 hs total; approx. 40 hs/ind
*Propithecus verreauxi* B	2 AF, 2 AM, 1 SAM, 1 SAF	November 2006–February 2007	240 hs total; approx. 40 hs/ind
*Eulemur rufus* x *collaris*	3 AF, 4 AM, 1 SAM, 3 SAF	March–July 2008	177 hs total; approx. 12 hs/ind

**Notes.**

AF adult females AM adult males SAF subadult females SAM subadult males

The physiological seasons (mating, pregnancy, birth and lactating/weaning seasons) influence the frequency of aggressive encounters. In *L. catta*, for example, aggression levels are highest—and conciliatory tendencies lowest—in the period around mating (Sclafani et al., 2012; Palagi & Norscia, 2014; Jolly, 1966). For this reason, observations were conducted in the period around mating for the three species (Table 1). The study groups shared part of their home range. The animals, habituated to human presence, were sexed and individually identified via facial-body features (Jolly, 1966).

The observations took place daily from dawn to dusk. The amounts of time devoted to the observations are reported in Table 1. We collected all avoidance submissive behaviors (walk away, cower, flee, and jump away; ethogram: Pereira & Kappeler, 1997) via focal animal sampling (Altmann, 1974). For submissive behaviors (total: 539 bouts; mean ± SE: 107.80 ± 46,38) we recorded actor’s and receiver’s identity. We collected data on dyadic agonistic encounters via all occurrence sampling (Altmann, 1974), and recorded (i) opponents, (ii) conflict type (decided versus undecided conflicts), and (iii) aggressive patterns (chasing, biting, and slapping). Decided conflicts (total: 957 bouts; mean ± SE: 191.40 ± 64.37) involve a clear winner, with an animal directing an aggressive behavior toward another individual (the victim), which flees or moves away either vocalizing or not. Undecided conflicts involve bidirectional aggressions from an individual to another, with both opponents either moving away or not from the location where aggressive behavior had occurred. Systematic data collection was preceded by training periods that lasted until the observations by the two-three observers matched in 95% of cases (Martin & Bateson, 1986). At the end of each training period, Cohen’s kappas (*k*) were higher than 0.70 for all three species (Kaufman & Rosenthal, 2009). For each behavioural category (submissive acts and aggressive events) we provide the kappa range (min–max) for all observer dyads: *k*_submissive_ = 0.71–0.75; *k*_aggression_ = 0.79–0.83. Group size and compositions, and observation periods and time are reported in Table 1.

Each day two observers randomly checked for the level of group cohesion (3–4 times a day) by recording the inter-individual spatial distance (more or less than 20 m) between group-members. *A posteriori* (after determining animals’ rank), we calculated the cohesion level at any given time as the number of individuals within 20 m from the dominant female over the total animal number of group members.

**Hierarchy linearity, steepness, triangle transitivity and statistical approach**

Hierarchy linearity was determined using Matman 1.0 (10.000 randomizations) by determining value of the Landau’s corrected linearity index *h*′ (which takes the number of unknown relationships and ties into account) and its statistical significance (de Vries, Netto & Hanegraaf, 1993; de Vries, 1995). When significant linearity was detected, dominance ranks were determined using the I&SI method and re-ordered to minimize inconsistencies and strengths of inconsistencies in dominance relationships (de Vries, 1998). The analysis was conducted on either aggression socio-matrices (based on dyadic decided conflicts) or avoidance socio-matrices.

The steepness was calculated from matrices of decided conflicts via Steepness 2.2 (Leiva & de Vries, 2011) and refers to the absolute slope of the straight line fitted to the normalized David’s scores plotted against the subjects’ ranks (de Vries, Stevens & Vervaecke, 2006). Normalized David’s scores (NDS) were calculated on the basis of a dyadic dominance index (Dij) in which the observed proportion of wins (Pij) is corrected for the chance occurrence of the observed outcome. The chance occurrence of the observed outcome is calculated on the basis of a binomial distribution with each animal having an equal chance of winning or losing in every dominance encounter (de Vries, Stevens & Vervaecke, 2006). The correction is necessary when, as in the case of our study groups, the interaction numbers greatly differ between dyads. We determined the NDS-based hierarchy by ranking the individuals according to their NDSs.

In order to assess between-group differences in hierarchical steepness we ran a covariance analysis (One Way Ancova; software: SPSS 20.0). We introduced NDSs as dependent variable; group ID as fixed factor; and rank attributed via NDS as covariate.

After entering data into text files (saved with “.dat” extension) we used the One Way Anova via randomization (Resampling Procedures 1.3 by David C. Howell; 10,000 permutations) to compare cohesion levels and the absolute differences of steepness values between adjacently ranked individuals across groups (*k* = 5). As post-hoc tests we applied the randomization test on two independent samples (between-group comparisons). Randomization procedures account for pseudo-replication (Manly, 1997) deriving from non-complete independence of data-points (namely when the same individual is included in more than one data bout).

By applying the correlation test via randomization we evaluated the correlation between the two hierarchies obtained via both binary dyadic dominance relationships (I&SI) (de Vries, Netto & Hanegraaf, 1993) and NDS values (Leiva & de Vries, 2011).

We calculated the proportion of transitive triangles relative to all triangles (*P_t_*) and the triangle transitivity metric (*t*_tri_) using the codes provided in Shizuka & McDonald (2012; supplementary material; errata corrige: Shizuka & McDonald, 2014). The codes to estimate triangle transitivity were applied on aggression sociomatrices using the package ‘statnet’ (Hankcock et al., 2003) in the R programming environment (R Development Core Team, 2011). To this purpose, data were entered in csv files.

## Results

Table 2 refers to aggression sociomatrices and shows all of the values related to binary dyadic relationships (I&SI), including Landau’s corrected index (*h*′), unknown and one-way relationships (%), and the Directional Consistency Index (DC). Table 3 shows the other results: linearity derived from avoidance sociomatrices (I&SI method) and outcomes from aggression sociomatrices (steepness, triangle transitivity, and consistency between NDS and I&SI hierarchies).

**Table 2:** Table referring to the presence of linearity and female dominance based on aggression sociomatrices. Landau’s corrected index (*h*′), level of probability, percentage of unknown and one-way relationships, and Directional Consistency Index (DC) are also reported.

Study groups	Female dominance	Linearity	Landau’s corrected index	Unknown relationships	One-way relationships	DC
*Lemur catta* A	yes^a^	yes^a^	*h*′ = 0.988 (*p* = 0.0001)	2.22%	75.56%	0.80
*Lemur catta* B	yes^a^	yes^a^	*h*′ = 0.686 (*p* = 0.0001)	20.51%	73.08%	0.95
*Propithecus verreauxi* A	yes^b^	yes^b^	*h*′ = 0.570 (*p* = 0.0350)	35.56%	37.78%	0.78
*Propithecus verreauxi* B	yes^b^	yes^b^	*h*′ = 0.886 (*p* = 0.0700)	26.67%	53.33%	0.91
*Eulemur rufus* x *collaris*	yes^c^	yes^c^	*h*′ = 0.509 (*p* = 0.0370)	30.91%	52.73%	0.67

**Notes.**

a Sclafani et al., 2012.

b Palagi, Antonacci & Norscia, 2008.

c Norscia & Palagi, 2011.

**Table 3:** Different dominance measures. Summary of values and/or level of probability referring to linearity (presence/absence) based on avoidance interactions (Landau’s corrected index, *h*′); steepness based on aggression sociomatrices; results of the correlation via randomization (coefficient *r* and probability); triangle transitivity based on aggression sociomatrices (*P_t_*: proportion of transitive triangles over the total; *t*_tri_: triangle transitivity metric); and cohesion around the dominant female. Steepness and triangle transitivity values are based on the matrices of aggressive interactions. The correlation via randomization refers to the correlation between hierarchies obtained via I&SI and normalized David’s scores (aggression sociomatrices).

Study groups	Linearity (avoidance interactions)	Steepness	Correlation	Triangle transitivity *P_t_*, *t*_tri_	Cohesion around the dominant female
LcA	yes (*h*′ = 0.751, *p* = 0.0012)	0.776 (*p* = 0.0001)	*r* = 0.99 (*p* < 0.001)	0.960, 0.839	0.8574 ± 0.0235
LcB	yes (*h*′ = 0.585, *p* = 0.0040)	0.460 (*p* = 0.0001)	*r* = 0.99 (*p* < 0.001)	0.996, 0.986	0.8036 ± 0.0347
PvA	no (*h*′ = 0.376, *p* = 0.2650)	0.278 (*p* = 0.0018)	*r* = 0.90 (*p* = 0.001)	0.840, 0.360	0.7209 ± 0.0202
PvB	no (*h*′ = 0.628, *p* = 0.2610)	0.444 (*p* = 0.0015)	*r* = 0.89 (*p* = 0.036)	1.000, 1.000	0.7321 ± 0.0249
Erxc	no (*h*′ = 0.350, *p* = 0.2520)	0.258 (*p* = 0.0024)	*r* = 0.83 (*p* = 0.003)	0.896, 0.582	0.5760 ± 0.0452

Avoidance-based matrices did not provide linear hierarchies for *Propithecus verreauxi* and *Eulemur rufus* x *collaris* groups. In contrast, the hierarchy of both *Lemur catta* groups remained linear and showed exclusive female dominance when based on avoidance-based matrices (Table 3). Yet in group A the ranking order in the avoidance based hierarchy was the same observed when the individuals were ordered on the basis of aggression sociomatrices (Table 4) whereas in group B nine individuals out of 13 changed their ranking position in the avoidance based hierarchy (compared to the aggression based hierarchy).

**Table 4:** Comparison of hierarchical orders assessed according to binary diadic dominance relationships (I&SI) and normalized David’s scores corrected for chance (NDS). Hierarchies of the different lemur groups, *Lemur catta* A (LcA) and B (LcB), *Propithecus verreauxi* A (PvA) and B (PvB), and *Eulemur rufus* x *collaris* (E), assessed according to binary diadic dominance relationships (I&SI) and normalized David’s scores corrected for chance (NDS). For all groups, the I&SI and NDS hierarchies deriving from aggression sociomatrices is reported. For the two groups of *L. catta*, the hierarchy obtained via I&SI methods from avoidance sociomatrices was linear. It is reported for LcB only, because for LcA the aggression and avoidance based hierarchies coincide. Grey blocks refer to females and white blocks to males. Females ranking under males are all subadult.

LcA_I&SI_	LcA_NDS_	LcB_I&SI-agg_	LcB_I&SI-av_	LcB_NDS_	PvA_I&SI-agg_	PvA_NDS_	PvB_I&SI-agg_	PvB_DS_	E_I&SI-agg_	E_NDS_
M	M	MY	MY	MY	P	MT	CA	BA	TS	OB
T2	T2	CS	CV	S	MT	P	BA	CA	BAPA	TS
TV	TV	S	CS	CS	GR	GR	BO	BRA	OB	BAPA
MS	MS	BI	S	BI	TB	UA	BRA	BO	PAL	PEN
T1	T1	CV	BI	BV	SCR	TB	BRO	BRO	PEN	PAL
BR	BR	BV	CSV	CV	UA	SCR	CL	CL	CM	FF
GR	GR	2T	BV	2T	OT	OT			MCN	CM
BO	BALL	CSV	2T	CSV	U	S			SX	ST
BALL	BO	P	PG	P	N	U			ST	SX
R	R	PG	P	PG	S	N			FF	MCN
		CO	CO	CO					FC	FC
		N	N	N						
		C	C	C						

**Figure 1:**
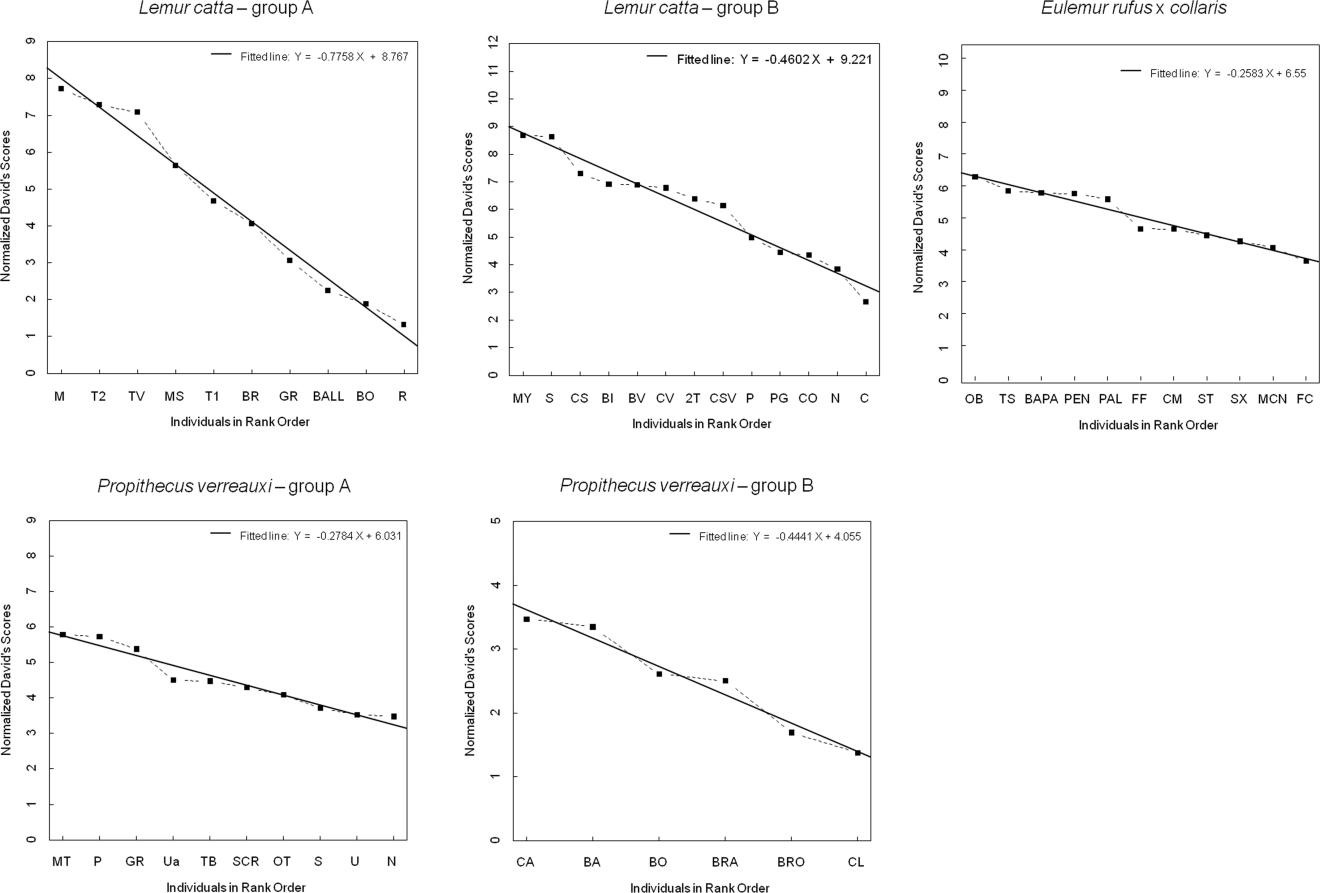
Normalized David’s scores plotted against rank order. The graph—output of Steepness 2.2—shows normalized David’s scores (corrected for chance, based on aggression sociomatrices) plotted against ordinal rank order (dashed black line), and the fitted line (black, solid line) for all the study groups (*Lemur catta* A and B, *Propithecus verreauxi* A and B, *Eulemur rufus* x *collaris*). The *Y* axis reports the Normalized David’s scores and the *X* axis reports the individuals of each group.

**Figure 2:**
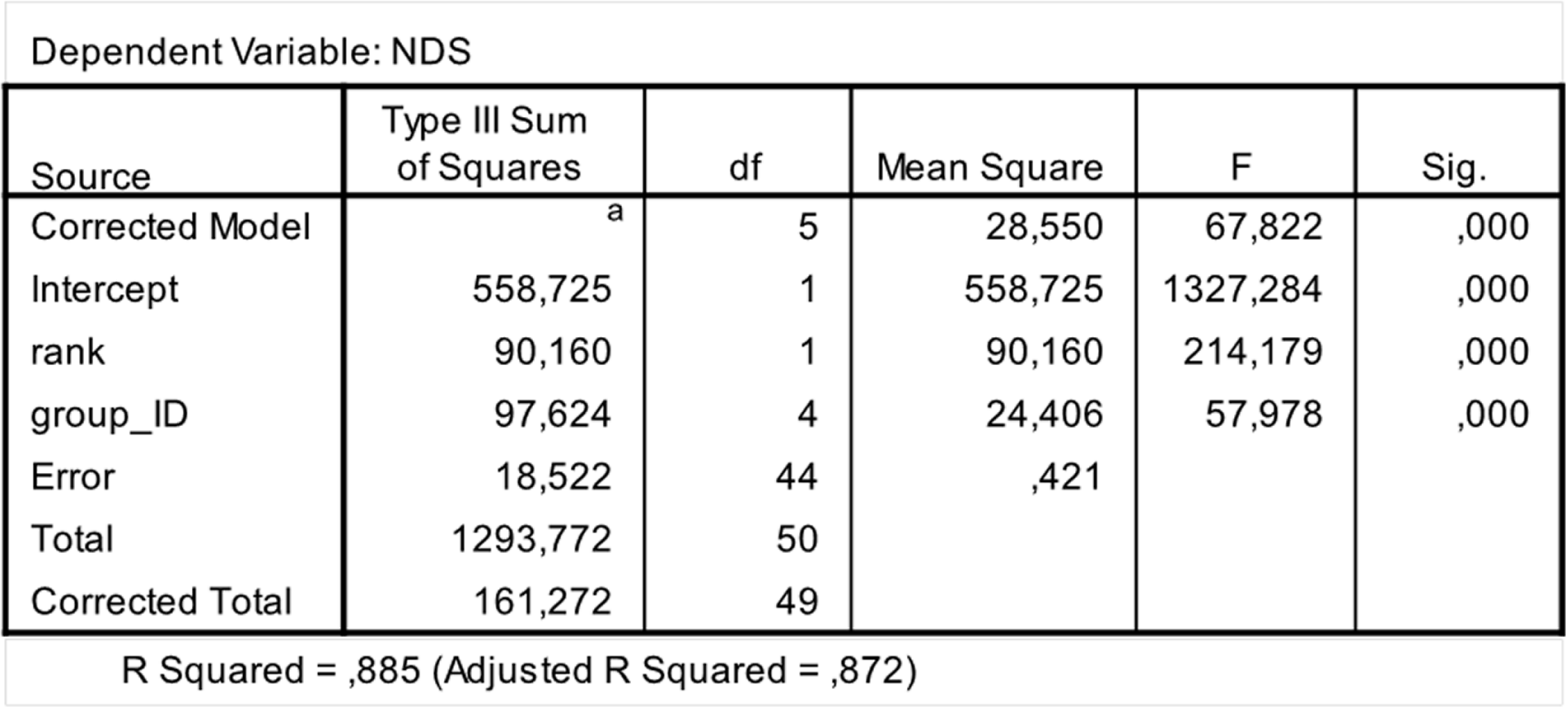
Results of the one-way analysis of covariance (ANCOVA). SPSS 20.0 output of the ANCOVA test run to check for between-group differences in hierarchical steepness. Dependent variable: Normalized David’s Scores (NDS); Fixed factor: Group ID; Co-variate: rank attributed by NDS.

**Figure 3:**
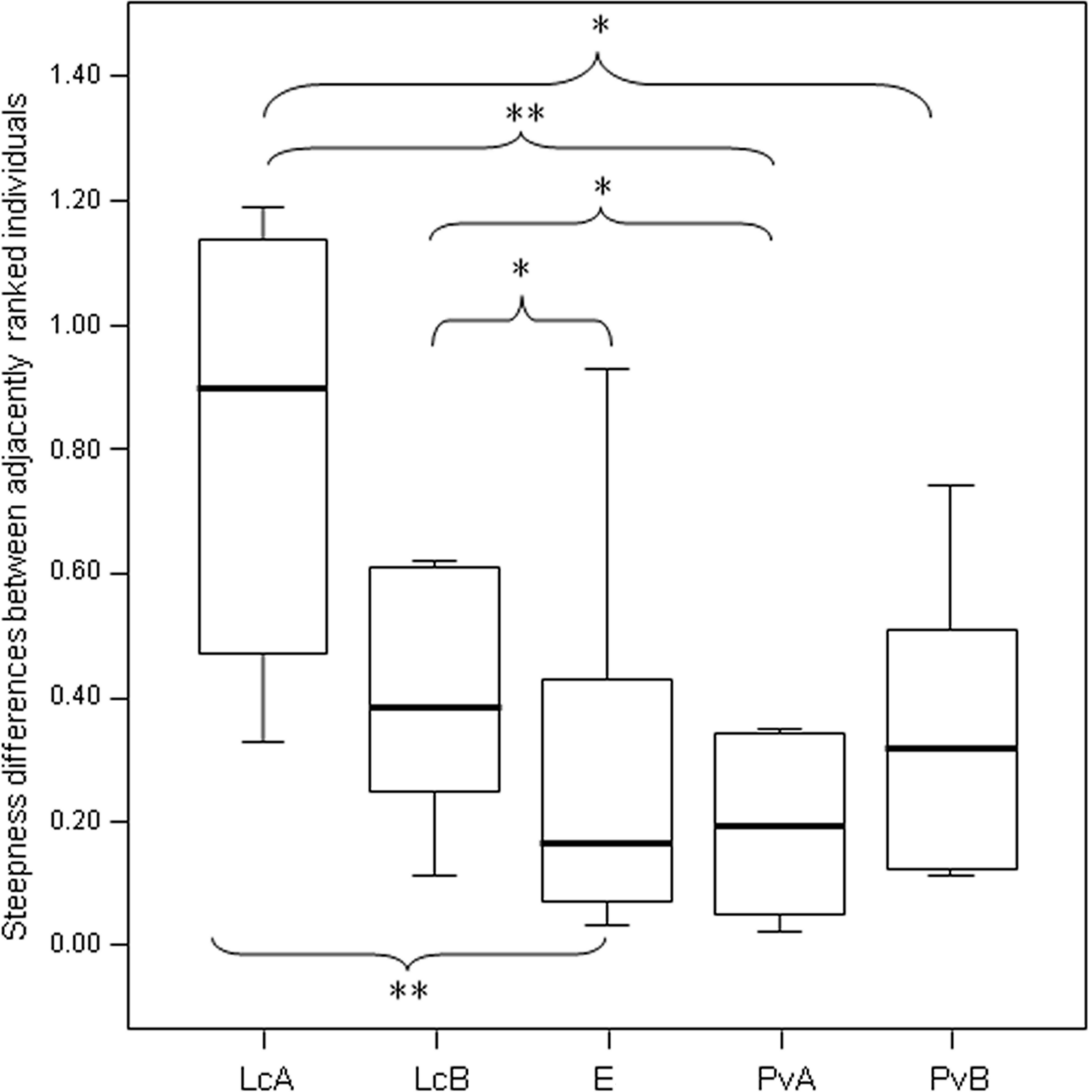
Difference in the group cohesion around the dominant across the five study groups. Box plot showing the comparison of the absolute differences of NDS values between adjacently ranked individuals of each group, across the five study groups (LcA, *Lemur catta* A; LcB, *Lemur catta* B; PvA, *Propithecus verreauxi* A; PvB, *Propithecus verreauxi* B; E, *Eulemur rufus* x *collaris*). Sample size (individuals): *n*_LcA_ = 9, *n*_LcB_ = 11, *n*_PvA_ = 9, *n*_PvB_ = 5, *n_E_* = 9. Results of the post-hoc randomization tests on two independent samples: PvB versus PvA: *t* = −0.704, *p* = 0.506; E versus PvB: *t* = 0.642, *p* = 0.545; E versus PvA; *t* = −0.068, *p* = 0.943; PvB versus LcB: *t* = 0.160, *p* = 0.281; PvA versus LcB; *t* = 2.150, *p* = 0.046; PvA versus LcA; *t* = 3.479; *p* = 0.005; PvB versus LcA; *t* = 2.225, *p* = 0.044; E versus LcB: *t* = 2.078, *p* = 0.049; E versus LcA: *t* = 3.462, *p* = 0.003; LcB versus LcA: *t* = 0.846, *p* = 0.413. (*) significant results (*p* < 0.05); (**) highly significant results (*p* < 0.01). Solid horizontal lines indicate medians; length of the boxes corresponds to inter-quartile range; thin horizontal lines indicate the range of observed values.

The steepness was highest for *Lemur catta* groups and lowest for the group of *Eulemur rufus* x *collaris*, with *Propithecus verreauxi* groups showing intermediate values (Table 3; Fig. 1). The steepness of hierarchies were significantly different across groups (One-way Ancova; results reported in Fig. 2). Also the absolute NDS differences between adjacently ranked individuals significantly differed across groups (One-way Anova via randomization *F* = 2.893, *df* = 4, *n*_LcA_ = 9, *n*_LcB_ = 12, *n*_PvA_ = 9, *n*_PvB_ = 5, *n*_Er_ = 10, *p* = 0.036; *n* indicates the number of inter-individual NDS differences corresponding to *n*−1 individuals per group). In particular, both groups of *L. catta* had significantly higher NDS differences than the *E. rufus x collaris* group. A group of *L. catta* (A)  also exhibited significantly higher NDS differences than both groups of *P. verreauxi*. In the other *L. catta* group (B), inter-individual NDS differences were significantly higher than those recorded for a group of *P. verreauxi* (A) but comparable to those shown by the other *P. verreauxi* group (B). Results of post-hoc randomization tests on two independent samples are shown in Fig. 3.

When—based on aggression sociomatrices—the individuals of each group were ordered according to both I&SI (based on binary dyadic dominance relationships) and their NDS (normalized David’s scores) (Table 4), the two resulting hierarchies correlated in all groups. The coefficient indicates that the group of *E. rufus* x *collaris* (Er_I&SI_ versus Er_NDS_: *r* = 0.83, *p* = 0.003) and the two *P. verreauxi* groups (PvA_I&SI_ versus PvA_NDS_: *r* = 0.90, *p* = 0.001; PvB_I&SI_ versus PvB_NDS_: *r* = 0.89, *p* = 0.036) had lower correlation levels than the two *L. catta* groups (LcA_I&SI_ versus LcA_NDS_: *r* = 0.99, *p* < 0.001; LcB_I&SI_ versus LcB_NDS_: *r* = 0.99, *p* < 0.001), with *E. rufus* x *collaris* showing the lowest correlation coefficient.

Triangle transitivity was highest for group B of *P. verreauxi* (*t*_tri_ =1) and for the two groups of *L. catta* (*t*_tri_ = 0.839; 0.986), and lowest for group A of *P. verreauxi* (*t*_tri_ = 0.360) and for the group of *E. rufus* x *collaris* (*t*_tri_ = 0.582) (Table 3).

**Figure 4:**
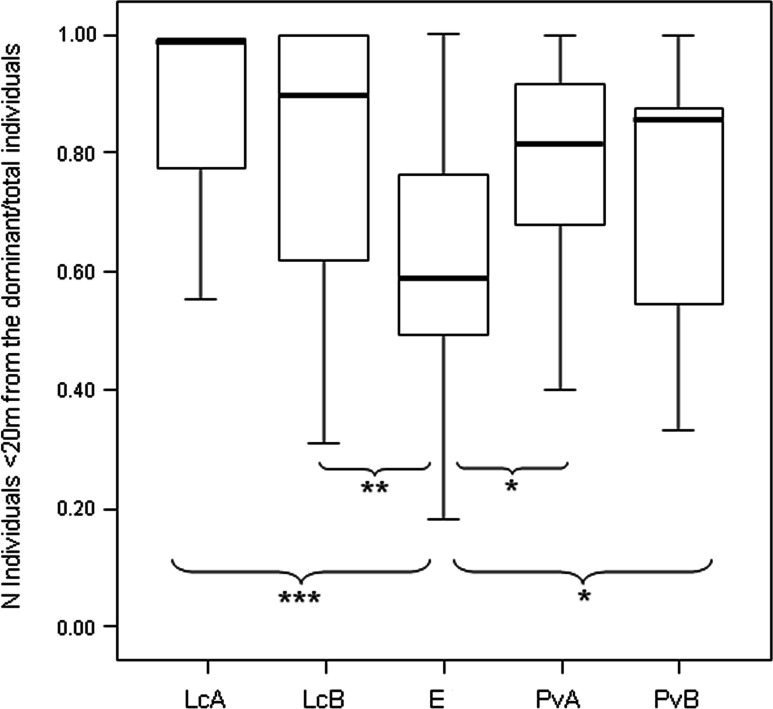
Difference in the group cohesion around the dominant across the five study groups. Box plot showing the difference in group cohesion around the dominant (proportion of individuals within 20 m from the dominant over the total animal number) across the five study groups (LcA, *Lemur catta* A; LcB, *Lemur catta* B; PvA, *Propithecus verreauxi* A; PvB, *Propithecus verreauxi* B; E, *Eulemur rufus* x *collaris*). Observational cohesion bouts for the five groups: *n*_LcA_ = 65, *n*_LcB_ = 40, *n*_PvA_ = 60, *n*_PvB_ = 77, *n_E_* = 34. Results of the post-hoc randomization tests on two independent samples: PvB versus PvA: *t* = −1.656, *p* = 0.101; E versus PvB: *t* = 2.101, *p* = 0.036; E versus PvA; *t* = 2.355, *p* = 0.021; PvB versus LcB: *t* = −1.800, *p* = 0.080; PvA versus LcB; *t* = −1.592, *p* = 0.121; PvA versus LcA; *t* = −1.581; *p* = 0.118; PvB versus LcA; *t* = −1.901, *p* = 0.058; E versus LcB: *t* = −2.995, *p* = 0.004; E versus LcA: *t* = −3.840, *p* < 0.001; LcB versus LcA: *t* = −0.326, *p* = 0.752. (^∗^) significant results (*p* < 0.05); (^∗∗^) highly significant results (*p* < 0.01); (^∗∗∗^) extremely significant results (*p* < 0.001). Solid horizontal lines indicate medians; length of the boxes corresponds to inter-quartile range; thin horizontal lines indicate the range of observed values.

After determining the dominant individual based on NDS hierarchy (aggression sociomatrices), we found that the proportion of group members packed around the dominant female at any given time (group cohesion) significantly differed across the five groups (Anova One-Way Randomization *F* = 7.173, *df* = 4, *n*_LcA_ = 65, *n*_LcB_ = 40, *n*_PvA_ = 60, *n*_PvB_ = 77, *n*_Er_ = 34, *p* < 0.001; *n* indicates the observational cohesion bouts). Post-hoc randomization tests on two independent samples revealed that group cohesion significantly differs between the *E. rufus* x *collaris* group and the groups of the other two species (statistical results are shown in Fig. 4).

## Discussion

As indicated in previous reports, all the groups under study are characterized by linear hierarchy and female dominance determined using aggression sociomatrices (Norscia & Palagi, 2011; Palagi & Norscia, 2011; Sclafani et al., 2012; Palagi, Antonacci & Norscia, 2008). Based on these characteristics only, we would conclude that similar dominance features apply to all groups. The multistep approach proposed here allows the drawing of a more detailed dominance profile of social groups, thus leading to a fine-grained distinction between them.

*Aggression- and submission-based hierarchy (step 1, prediction 1)*

The first step of our approach allows the detecting of how hierarchy linearity is established (via either overt aggressions or avoidance, or both) in different social groups. We used avoidance, not elicited by any aggressive behavior but indirectly correlated with the outcome of decided agonistic encounters, to verify whether it provides the same dominance structure (linearity, female dominance) obtained via aggression sociomatrices. The two *L. catta* groups stand out because they maintained linearity whereas the other groups did not (Tables 2 and 3; Prediction 1 partly confirmed). Contrary to the prediction, *P. verreauxi* groups did not have a linear hierarchy based on avoidance probably because the use of avoidance behavior does not reflect the use of formalized submissive chatters and/or because the hierarchical relationships are more relaxed (Kappeler, 1999; Norscia, Antonacci & Palagi, 2009). In *L. catta* groups the linearity of avoidance based hierarchy derives from the highest frequency of unidirectional dyadic avoidance behavior in *L. catta* groups and it can indicate greater acceptance of the inferior social rank to dominants by subordinates (deference), greater intolerance by dominants to subordinates, or both. We define hierarchy here as aggression-based if it is exclusively unveiled by overt aggressions and submission-based if its detection does not necessarily depend on an arena of aggressive encounters. According to this definition, linear hierarchy is both aggression- and submission-based in *L. catta* groups and aggression-based in *P. verreauxi* and *E. rufus* x *collaris* groups.

Previous works have reported the coexistence of more than one hierarchy at the same time, often behavior dependent. Richard (1974) in *Propithecus verreauxi* detected no consistent correlation between the rank of individuals ordered according to the criterion of priority of access to food (feeding hierarchy) and their rank established according to the frequency of aggression, the direction and frequency of grooming, or preferential access to females during the mating season. Alvarez (1975) observed that hierarchy in squirrel monkeys (*Saimiri sciureus*) varied from quasi-linear to circular, depending on the behavioral patterns considered for rank assessment (approaching, following, withdrawing, and genital inspection). de Waal & Luttrell (1985) described behavior dependent hierarchies and distinguished between real and formal dominance relationships in rhesus macaques (*Macaca mulatta*), with the former depending on agonistic encounters and the latter only depending on unidirectional and context independent signals (de Waal, 1982; de Waal, 1986). Similarly, a troop of ringtailed lemurs (group B) showed behavior dependent hierarchy. In fact, in this group the ranking order obtained via avoidance sociomatrices differed from the ranking order generated by aggression sociomatrices (Table 4). Even though the same leader and exclusive female dominance were maintained in both aggression and avoidance based hierarchies, many individuals possessed a different position in the two hierarchies (Table 4). Thus, the power discrepancy perceived by individuals (asymmetry derivable from avoidance behavior) does not necessarily go in tandem with the asymmetry established via aggressive interactions.

The difference observed in the ranking order and linearity level is also related to the lower number of avoidance events compared to decided agonistic encounters recorded in the study groups, which is in line with the fact that in the period around mating aggression rates are higher than in other periods in wild lemurs (*L. catta*: Jolly, 1966; Gould & Ziegler, 2007; *P. verreauxi*: Brockman, 1998; Brockman et al., 1998; *Eulemur rufus*: Ostner, Kappeler & Heistermann, 2002).

The twofold approach presented here, which considers both submissive and aggressive interactions, unravels divergences between perceived and aggression based power asymmetry in species that are classically considered as despotic (e.g., baboons, Rowell, 1967; mandrills, Wickings & Dixson, 1992; wolves, Cordoni & Palagi, 2008).

*Shallow versus steep hierarchy (step 2, prediction 2)*

The second step allows separating social groups according to hierarchy steepness. When steepness is used to evaluate the dominance structure based on aggression sociomatrices, other inter-group differences—not revealed by linearity—emerge. The different groups indeed differed in their hierarchical steepness (Fig. 1; Fig. 2). The comparison of dyadic NDS values across groups allowed segregating the *L. catta* groups from the group of *E. rufus* x *collaris*, with ringtailed lemur groups showing the steepest hierarchy gradient. Conversely, *P. verreauxi* groups and the *E. rufus* x *collaris* group showed similar steepness levels. Prediction 2 is overall supported but it is worth remarking that the differences in steepness levels between *L. catta* and *P. verreauxi* groups varied depending on the groups considered (Fig. 3). This situation is in line with the observations of Balasubramaniam et al. (2012) on different macaque species (ranked from grade 1 to 4 depending on their tolerance levels). The authors observed that steepness measures were more continuous than other measures (e.g., counter-aggression) and did not fully match the species separation into different tolerance grades. Consequently, they noted that different aspects of social style may display somewhat different patterns of variation across species, and that covariation between even closely related measures may be imperfect (Balasubramaniam et al., 2012).

*Weakly versus strongly consistent hierarchy (step 3, prediction 3)*

The third step allows differentiating groups according to another property: hierarchy consistency. By way of both I&SI (binary dyadic dominance relationships) and NDS (normalized David’s scores corrected for chance) methods, all adult females outranked adult males in all study groups, thus confirming the exclusive dominance of females over males (Table 4). Overall the I&SI and NDR correlated in all groups and were quite consistent, even if the top ranking female remained the same only in the two ringtailed lemur groups (Table 4). Therefore, the hierarchy appears to be more rigid in *L. catta*, apparently sealing off individual movement within the hierarchy (cf. Tables 2–4; Prediction 3 confirmed).

As specified above, different ranking positions in the same group can be observed for the same individuals when they are context or behavior dependent (e.g., present study, aggression- versus submissive-based hierarchy in *Lemur catta*; Richard, 1974; Alvarez, 1975; de Waal & Luttrell, 1985). In the case of our study groups, the two different hierarchical arrangements, especially detectable in sifaka and brown lemurs (Table 4), are both generated by the same aggression sociomatrices, through the application of different analyses: I&SI which focuses on the direction of aggression asymmetry; and NDS, which also considers the extent of aggression asymmetry and dyadic encounter probability. It is the quantitative approach itself that reveals two different hierarchy properties.

*Less versus more cohesive hierarchy (step 4, prediction 4)*

The measure of triangle transitivity (excluding dyads without interactions; *t*_tri_; Shizuka & McDonald, 2012) provides a further (and different) clustering of our study groups, with a group of *P. verreauxi* (B) and the two groups of *Lemur catta* showing the highest transitivity levels, and *E*. *rufus* x *collaris* and a group of *P. verrauxi* (A) the lowest levels (Table 3). The lower transitivity values observed for *E*. *rufus* x *collaris* and a group of *P. verrauxi* (A) (compared to the other study groups) correspond to weaker group cohesion around the dominant (Table 3), even if the groups of *L. catta* and *P. verreauxi* did not significantly differ in the cohesion levels (Fig. 4) (Prediction 4 only partially confirmed). On the other hand, the highest levels of triangle transitivity in *L. catta* just confirm the rigid ranking order, corresponding to the highest group packing around the dominant. The top triangle transitivity value was recorded for the group B of *P. verreauxi*. Because the number of known relationships in this group is smallest (Table 2), the likelihood of finding a relatively large *t*_tri_ value in this group is larger than in the other groups where the numbers of known relationships are much larger. Shizuka & McDonald (2012) reported that the proportion of zero dyads is positively correlated with group size and ten out of twelve groups of six individuals included in their study showed maximum triangle transitivity (*t*_tri_ = 1). The tightest bonds linking group members in *L. catta* and *P. verreauxi* (Fig. 4; Table 3) fit with previous literature, which refers to ring-tailed lemur and sifaka as cohesive units (Jolly, 1966; Richard, 1985). In a behavioral ecology perspective, the high group dispersion observed in brown lemurs is consonant with their habitat use pattern. At Berenty, they tend to extend resource exploitation in terms of diet variety (Jolly et al., 2000; Pinkus, Smith & Jolly, 2006), amount of food intake (Simmen, Hladik & Ramasiarisoa, 2003), temporal activity (Donati et al., 2009) and ranging patterns (Tanaka, 2007). The higher is the spatial dispersion of an animal group, the lower is the level of contact opportunities. This can explain, at least in this group, the higher percentage of unknown relationships (Table 2) leading to less transitive relationships. Another possibility is that the observed inter-species variations in dominance property may emerge not just from ecological, but also from phylogenetic constraints. It is not the prerogative of this study to test the explanatory models put forward by sociobiologists that posit variation in dominance relationships (e.g., Lewis, 2002; Hemelrijk, Wantia & Isler, 2008; Wilson, 2000) but future work should attempt to do so.

In our case, it is possible to state that *L. catta* and *P. verreauxi* groups show more cohesive hierarchies than *Eulemur rufus* x *collaris*, meaning that in the two former species a higher proportion of group members is found close to the dominant females (within 20 m) at any given time. However, the level of relationship transitivity is higher in *Eulemur rufus* x *collaris* than in one group of *P. verreauxi* (Table 3). We could interpret this very last result (*Eulemur rufus* x *collaris* not showing the lowest transitivity values of all) as a result biased by the presence of non-interacting diads. In fact, if patterns of non-interactions are not random because some dyads do not actually interact, the formation of transitive versus cyclic triangles can be skewed (Shizuka & McDonald, 2012). Instead, we speculate that the comparison between triangle transitivity and linearity provides the hierarchy assessment with an added value because it suggests that in the core group of *Eulemur rufus* x *collaris* (composed by individuals that actually interact with each other) relationships are more transitive than it appears by considering linearity alone. The observation of the different cohesion levels helps in explaining this difference by reinforcing the idea that hierarchy is less cohesive in the brown lemur group because the presence of non interacting dyads (informed by the weak group cohesion around the dominant) does not affect transitivity (non interacting dyads excluded) as much as it affects linearity (non interactive dyads included).

## Conclusions

We applied a four-step approach on a large database gathered, with the same observation protocol, on five wild multimale-multifemale lemur groups. The groups shared the same habitat, and part of the home range, and they were all characterized by linear hierarchy and female dominance (Norscia & Palagi, 2011; Palagi & Norscia, 2011; Sclafani et al., 2012; Palagi, Antonacci & Norscia, 2008). This information alone would lead to conclude that their dominance profile is alike. We used different measures (linearity, steepness, consistency, triangle transitivity and group cohesion) to determine whether group hierarchy was (i) aggression or submission based; (ii) shallow or steep; (iii) weakly or strongly consistent; and (iv) more or less cohesive.

Lemur groups showed different types of similarities and dissimilarities depending on the measure used. For example, dominance relationships of the *E. rufus* x *collaris* group and *P. verreauxi* groups are similar according to the steepness levels but can be different according to triangle transitivity and group cohesion. *L. catta* groups are more similar to *P. verreauxi* groups in terms of group cohesion, but not necessarily in terms of triangle transitivity or steepness. *Lemur catta* and *E. rufus* x *collaris* largely differ in steepness and level of linearity. Overall, *L. catta* groups show a linear, steep, consistent and highly transitive and cohesive hierarchy. *P. verreauxi* groups show a linear, moderately steep and consistent hierarchy, with variable levels of triangle transitivity and cohesiveness. *E. rufus* x *collaris* shows a linear but shallow and inconsistent hierarchy, with lower (but not lowest) levels of transitivity and scarce cohesiveness (but more groups should be considered to accurately assess this last property).

In conclusion, the use of the same method (I&SI) applied to different behavioral databases (aggression/avoidance), and different methods (normalised David’s scores, binary dyadic dominance relationships, triangle transitivity) applied to the same behavioral database (aggression sociomatrices), resulted in different dominance outlines relative to the same study subjects. The use of different methodological approaches is important because each single measure has its own limits: for example, linearity does not appreciate the different extent of power asymmetry between individuals, steepness can suffer from the presence of zero dyads, triangles of individuals may not be fully independent because each triangle within a social network can share nodes (individuals) and ties (connections) with other triangles (Flack & de Waal, 2004; de Vries, Stevens & Vervaecke, 2006; Wasserman & Katherine, 1994). Finally, a multiple analytical approach can lead to a more in-depth description of dominance profile, which is a multilevel concept combining many aspects of social dominance.

## Supplemental Information

10.7717/peerj.729/supp-1 Data S1 Database used for the analyses
